# At Risk and Underprepared: Training Gaps in Physical Mobility Support Among Aging Informal Caregivers

**DOI:** 10.1093/geroni/igaf122.3717

**Published:** 2025-12-31

**Authors:** Rodney Weir, Heather Renter, Olivia Brunner, Connor Lynch, Juliana Roller, Chloe Tomasic

**Affiliations:** Western Michigan University, Kalamazoo, Michigan, United States; Heritage Community of Kalamazoo, Kalamazoo, Michigan, United States; Western Michigan University, Kalamazoo, Michigan, United States; Western Michigan University, Kalamazoo, Michigan, United States; Western Michigan University, Kalamazoo, Michigan, United States; Western Michigan University, Kalamazoo, Michigan, United States

## Abstract

This qualitative research project explored the educational needs of informal, unpaid caregivers who assist individuals with chronic illnesses in performing physical mobility tasks. Many caregivers find themselves responsible for complex physical tasks which may include transfers, lifting, and the use of adaptive equipment. Previous research has documented that informal caregivers often perform such tasks with little or no training or support. This lack of preparation can lead to caregiver injury, emotional stress, and compromised patient safety. To investigate these challenges, researchers conducted focus group interviews with seven informal caregivers. Using a grounded theory approach, transcripts were analyzed to identify recurring themes related to caregiver education and training. Five key themes emerged: (1) “Making Do,” where caregivers improvised with equipment due to insufficient instruction; (2) emotional and psychological stress stemming from low confidence and self-efficacy; (3) physical strain and lack of self-care; (4) limited access to training and essential resources; and (5) difficulty navigating the healthcare system. The findings highlight a critical gap in caregiver education, particularly in the safe and effective use of mobility aids and techniques. Physical therapists, with their expertise in adaptive equipment and movement strategies, are well-positioned to address these gaps by providing targeted education and support. Enhancing caregiver training not only improves safety and care quality but also supports caregivers’ well-being and sustainability in their roles. Future research should examine the needs of older caregivers and the potential for physical therapy to extend its educational role in community-based care settings.

